# Supplementary material for: Applicability Domain of Active Learning in Chemical Probe Identification: Convergence in Learning from Non-Specific Compounds and Decision Rule Clarification
Source: Molecules. 2019 Jul 26;24(15):2716. doi: 10.3390/molecules24152716 (PMC6696588; doi:10.3390/molecules24152716)

*Supplementary figures*

# Applicability domain of active learning in chemical probe identification: convergence in learning from non-specific compounds and decision rule clarification

**Ahsan Habib Polash [1,2], Takumi Nakano [1], Shunichi Takeda [2] and J.B. Brown [1,*]**

[1] Kyoto University Graduate School of Medicine, Department of Molecular Biosciences, Life Science Informatics Research Unit; Kyoto, Sakyo, Yoshida, Konoemachi, 606-8501, Kyoto Japan
[2] Kyoto University Graduate School of Medicine, Department of Radiation Genetics; Kyoto, Sakyo, Yoshida, Konoemachi, 606-8501, Kyoto Japan
* Correspondence: jbbrown@kuhp.kyoto-u.ac.jp; Tel.: +81-75-753-9515 (J.B.)

Received: date; Accepted: date; Published: date

**Figure S1a.** (a,b) Experiments with physicochemical properties and either amino acid or tripeptide frequency protein descriptors. (c) Experiments with combination physicochemical and ECFP descriptors, using dipeptide frequency.

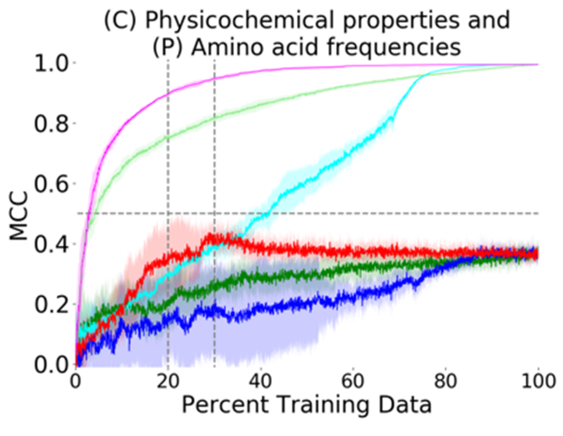


**(a)**

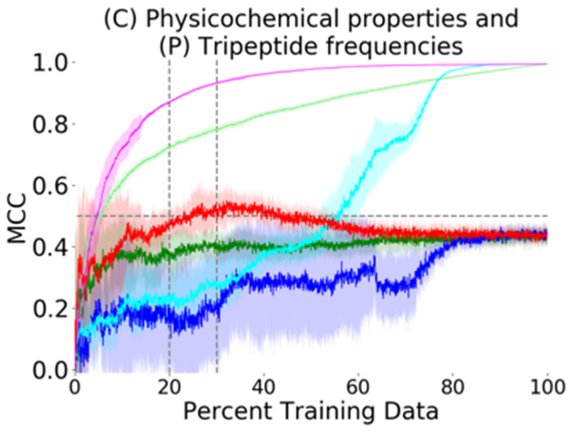


**(b)**

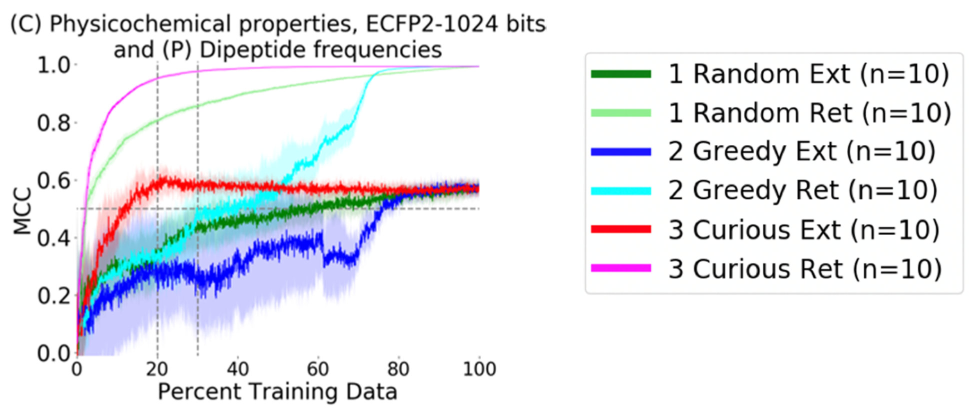


(c)

**Figure S1b.** Dipeptide frequency **(b)** with ECFPr2-1024 results in higher performance compared to the identity protein descriptor **(a)**.

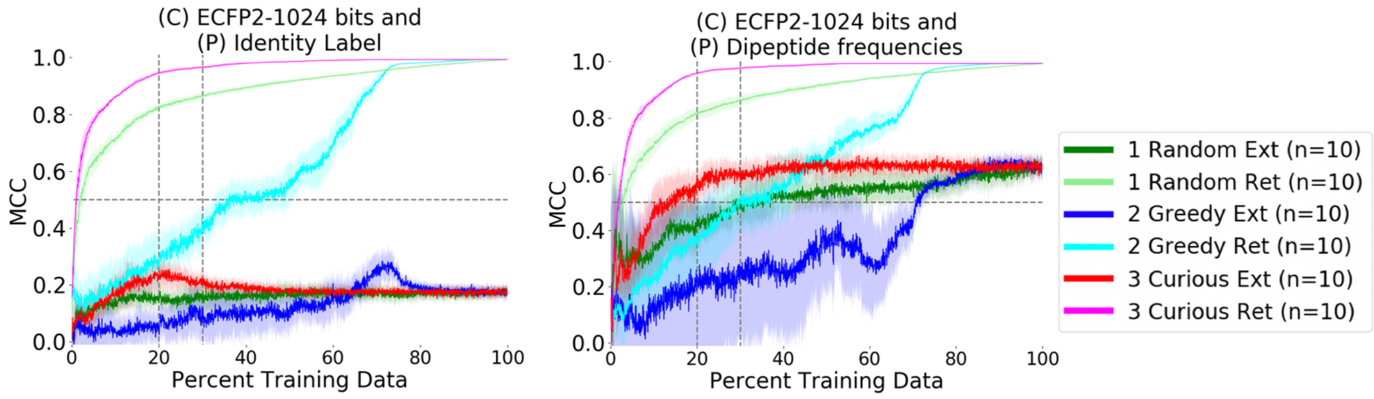


**(a)** **(b)**

**Figure S1c.** ECFPr2-4096 bits **(b)** in combination with dipeptide frequency results in equal or slightly better performance compared to the ECFPr2-512 bit fingerprints **(a)**, and a notable improvement over ECFPr2-1024 bit fingerprints.

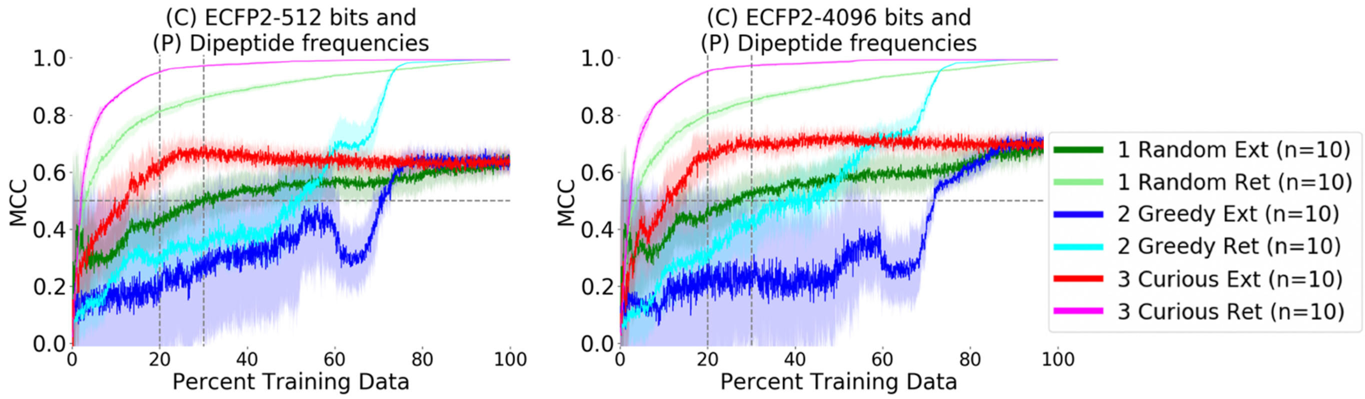


**(a)** **(b)**

**Figure S1d**. CATS2D descriptors with dipeptide frequency **(b)** display better performance compared to identity protein descriptors **(a)** and physicochemical-based descriptors.

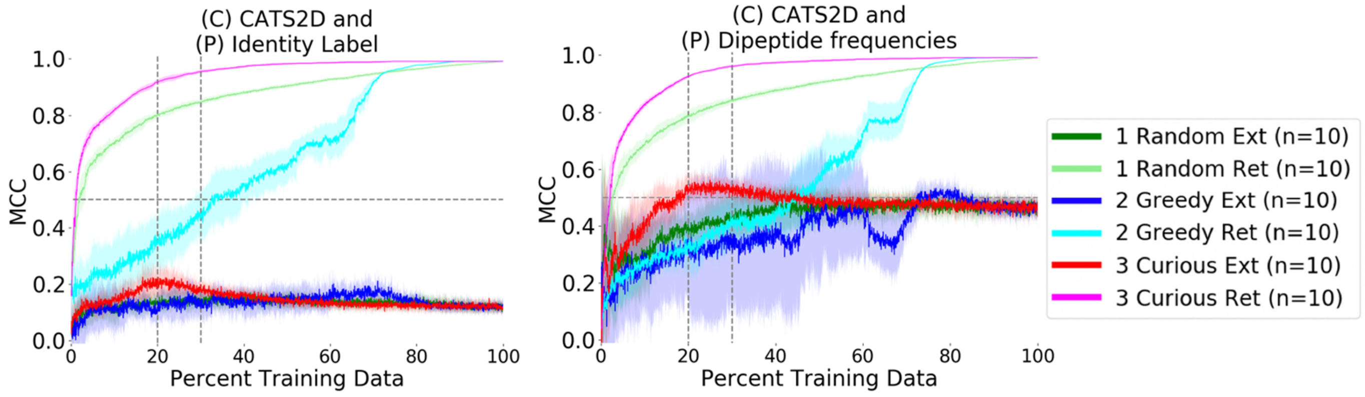


**(a)** **(b)**

**Figure S1e**. CATS2D descriptors with tripeptide frequency and tetrapeptide frequency display better performance on the external set compared to dipeptide frequency, amino acid frequency and identity protein descriptors.

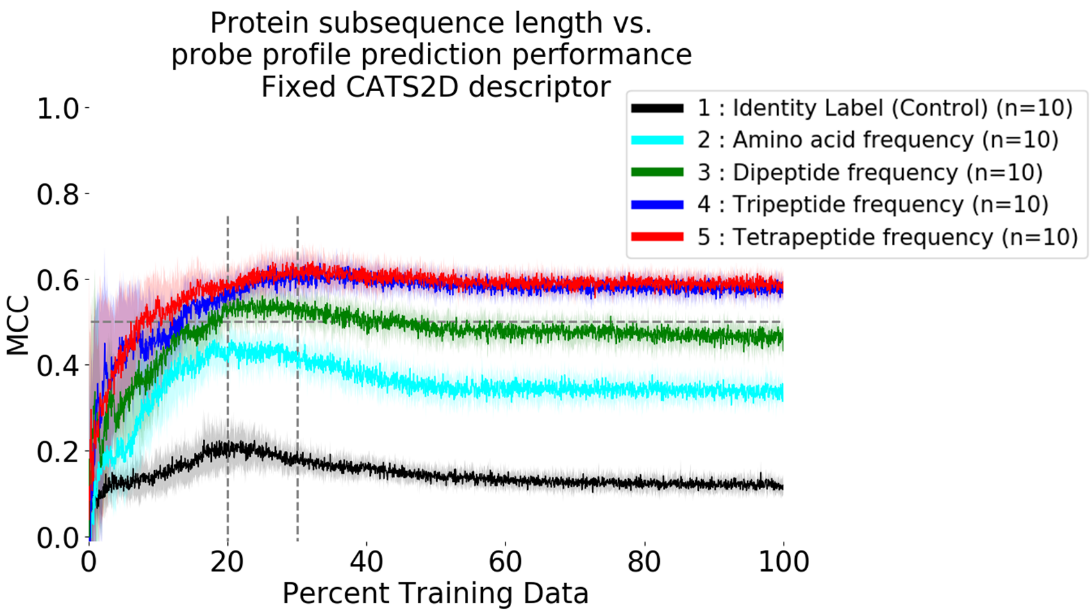


**Figure S2. (a)** Active projection for pChem-dipeptide description of ligand-target interactions; **(b)** pChem-tripeptide frequency descriptor-based model evolution.

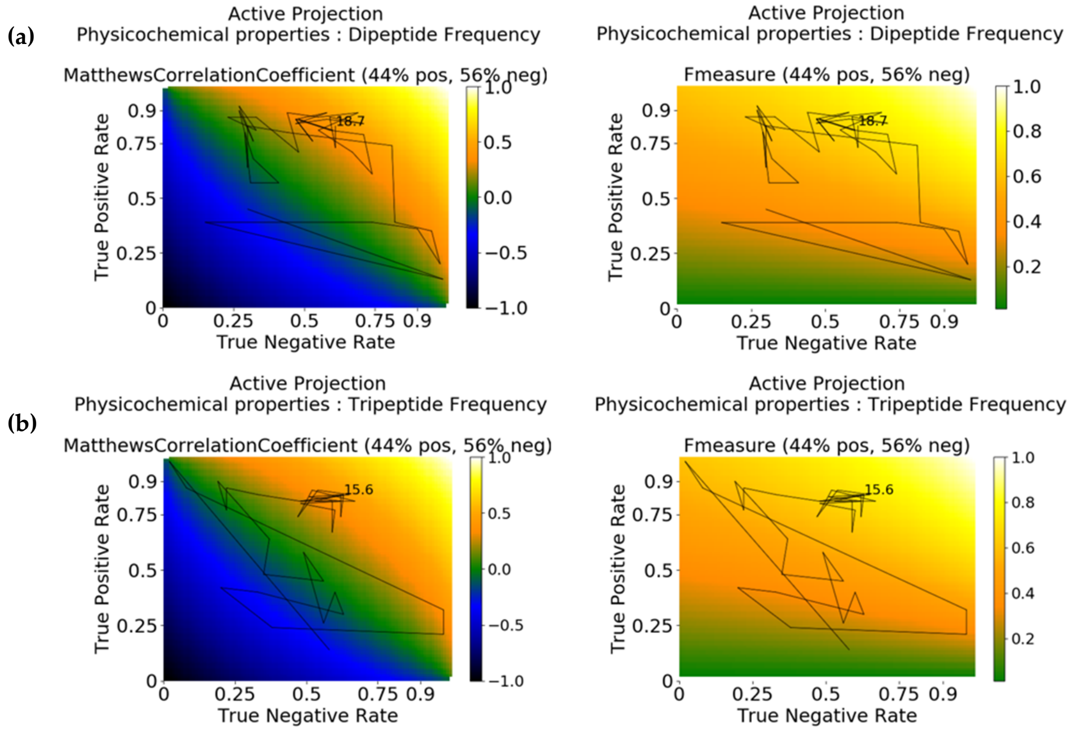

**Figure S3a**. Feature weight time series shows the evolving relative weights of each CATS2D-dipeptide descriptor used during model construction. Compound descriptors are the highest weighted features, whereas protein dipeptide frequencies are less weighted yet still non-trivial.

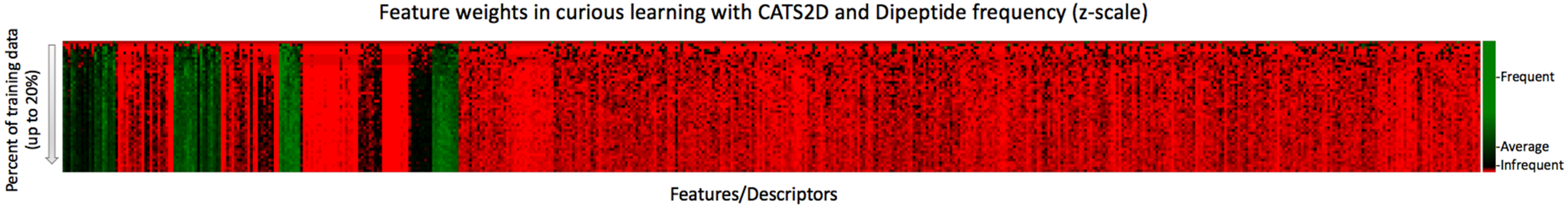


**Figure S3b.** Feature weight analysis of pChem-identity **(a)** and pChem-dipeptide experiments **(b)**.

(a)

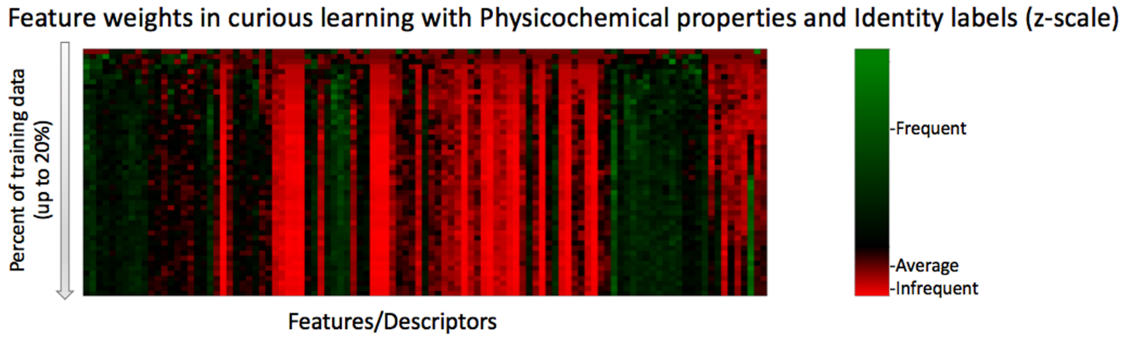


(b)

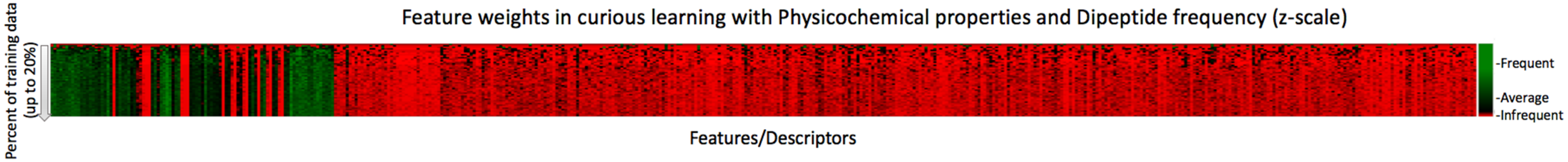

**Figure S4a**. A diagram showing how the protein tripeptide descriptors unique to a protein appeared in a specific decision tree. Many tripeptide rules could be found in multiple trees.

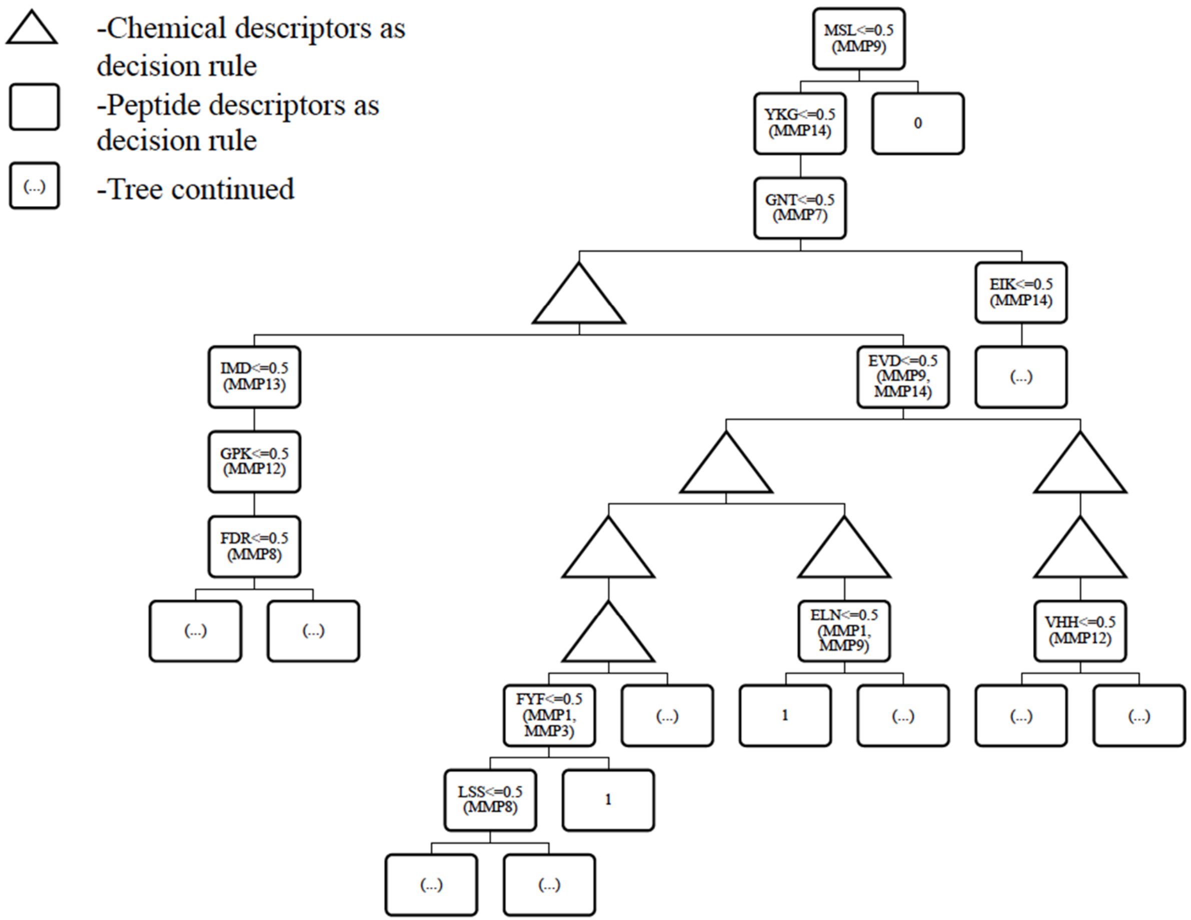

**Figure S4b**. A decision tree built on 20% of the training data with a predictive ability of MCC=0.50, F1=0.73 on the external probe dataset.

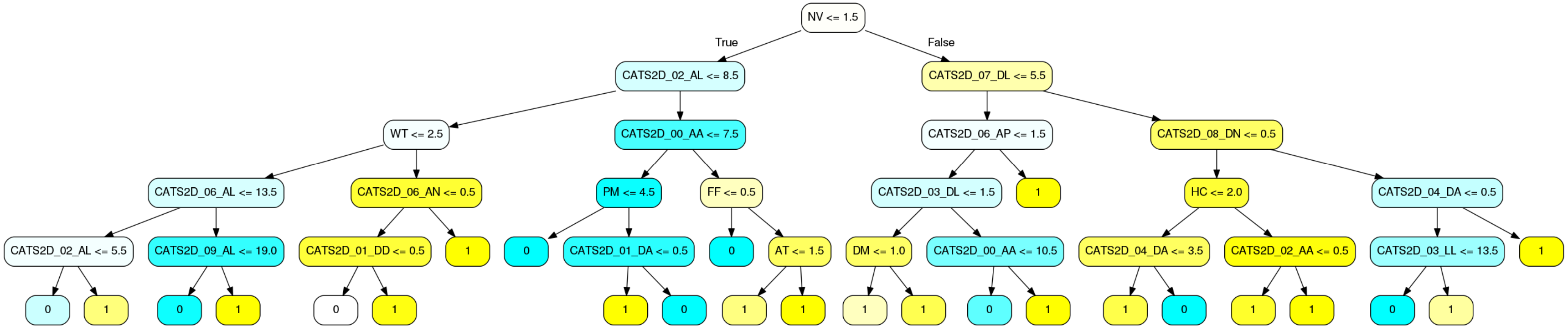

Supplement: Supplementary file 1 [file molecules-24-02716-s001.pdf]
